# The Expanding Burden of Neurodegenerative Diseases: An Unmet Medical and Social Need

**DOI:** 10.14336/AD.2024.1071

**Published:** 2025-11-04

**Authors:** Shu Wang, Yin Jiang, Anchao Yang, Fangang Meng, Jianguo Zhang

**Affiliations:** ^1^Department of Neurosurgery, Beijing Tiantan Hospital, Capital Medical University, Beijing 100070, China.; ^2^Department of Functional Neurosurgery, Beijing Neurosurgical Institute, Capital Medical University, Beijing 100070, China.; ^3^Beijing Key Laboratory of Neurostimulation, Beijing 100070, China

**Keywords:** Neurodegenerative disease, Alzheimer’s disease, dementia, Parkinson’s disease, burden of disease, global health

## Abstract

Neurodegenerative diseases, particularly Alzheimer’s disease and other dementias as well as Parkinson’s disease, are emerging as profoundly significant challenges and burdens to global health, a trend highlighted by the most recent Global Burden of Disease (GBD) 2021 studies. This growing impact is closely linked to the demographic shift toward an aging population and the potential long-term repercussions of the COVID-19 pandemic, both of which have intensified the prevalence and severity of these conditions. In this review, we explore several critical aspects of this complex issue, including the increasing global burden of neurodegenerative diseases, unmet medical and social needs within current care systems, the unique and amplified challenges posed by the COVID-19 pandemic, and potential strategies for enhancing healthcare policy and practice. We underscore the urgent need for cohesive, multidisciplinary approaches across medical, research, and policy domains to effectively address the increasing burden of neurodegenerative diseases, thereby improving the quality of life for patients and their caregivers.

## 1. Introduction

Neurodegenerative diseases, such as Alzheimer’s disease (AD) and other dementias, as well as Parkinson’s disease (PD), are increasingly recognized as major public health concerns with profound implications for individuals, families, and societies worldwide [1-3]. The increasing prevalence of these conditions is closely tied to the global demographic transition, characterized by an expanding elderly population [4-7]. This demographic shift, coupled with the potential long-term effects of the COVID-19 pandemic, has increased the urgency of addressing the growing burden of neurodegenerative diseases [8, 9]. The Global Burden of Disease (GBD) 2021 studies [4, 10] and the GBD 2021 study for Nervous System Disorders [11] have recently been updated to provide the latest evidence to guide advocacy and future efforts. The burden of conditions affecting the neurological system is the top-ranked contributor to global disability-adjusted life-years (DALYs) and years lived with disability (YLDs) [10, 11]. Crucially, global DALY counts of neurological disorders increased by 18.2% (95% uncertainty interval [UI] 8.7-26.7) from 1990 to 2021 and affected more than 40% of the global population [11]. One of the major contributors to the increasing burden of neurological conditions is the aging of population structures across the globe [4], which increases the prevalence and burden of neurodegenerative diseases [12]. These diseases constitute a significant portion of the global disease burden, affecting millions and imposing substantial medical, social, and economic costs [6, 11].

The impact of neurodegenerative diseases extends beyond individual patients, affecting their caregivers, who often face emotional, physical, and financial strains [13, 14]. Moreover, healthcare systems across different countries and territories are under increasing pressure to provide adequate, accessible, and effective care for older people with neurodegenerative diseases, which in many cases remains insufficient because of limited resources, a lack of specialized knowledge, and inadequate policy frameworks and strategies [6, 15-19]. In light of these challenges, there is a critical need for a comprehensive review that highlights the current state and future projections of neurodegenerative diseases, identifies the unmet needs of existing care models, and proposes strategies for improvement [6].

**Figure 1.**
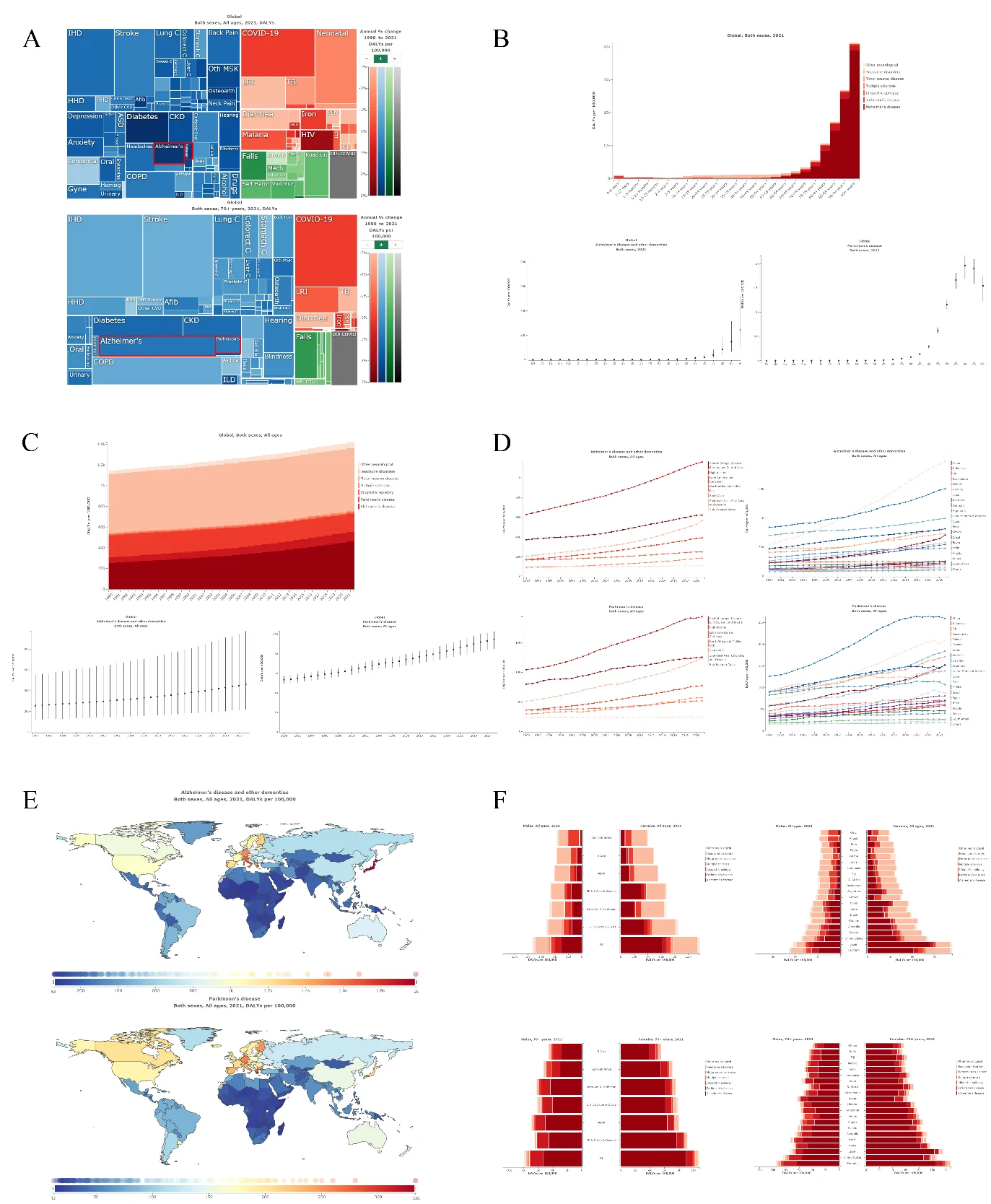
**Current global burden of neurodegenerative diseases, such as Alzheimer’s disease and other dementias, and Parkinson’s disease**. Global burdens (A), age profiles (B), trends (C and D), and geographical distributions (E and F) of disability-adjusted life-years (DALYs) of neurodegenerative diseases, such as Alzheimer’s disease and other dementias, and Parkinson’s disease according to the Global Burden of Disease (GBD) studies 2021. Sub-Saharan Africa, Sub-Saharan Africa; S Asia, South Asia; MENA, North Africa, and the Middle East; SE & E Asia & Oceania, Southeast Asia, East Asia, and Oceania; Latin Am & Caribbean, Latin America and the Caribbean; C & E Europe & C Asia, Central Europe, Eastern Europe, and Central Asia; HI, high income. Regions were stratified based on the GBD super-regions, which group countries and territories that share similar geographic characteristics, epidemiological profiles, and causes of death. All panels were drawn via GBD. Compare Data Visualization tools with data from GBD 2021 estimates. *Source: Institute for Health Metrics and Evaluation, University of Washington. Used with permission. All rights reserved.*

In this review, we aim to provide an in-depth analysis of the multifaceted issues surrounding the increasing burden of neurodegenerative diseases [10, 11, 20]. We will examine the latest epidemiological data and forecasted trends, discuss current unmet medical and social needs, and explore the specific challenges introduced or exacerbated by the ongoing and post-COVID-19 context [8, 10, 11, 21]. Furthermore, we address the gaps in medical and social support, emphasizing the need for innovative solutions and integrated approaches across various sectors [5, 18]. By doing so, we seek to contribute to the development of more robust and responsive healthcare policies and practices, ultimately aiming to enhance the quality of life for those affected by neurodegenerative diseases and their caregivers [22, 23].

## 2. Increasing global burden of neurodegenerative diseases

**Figure 2.**
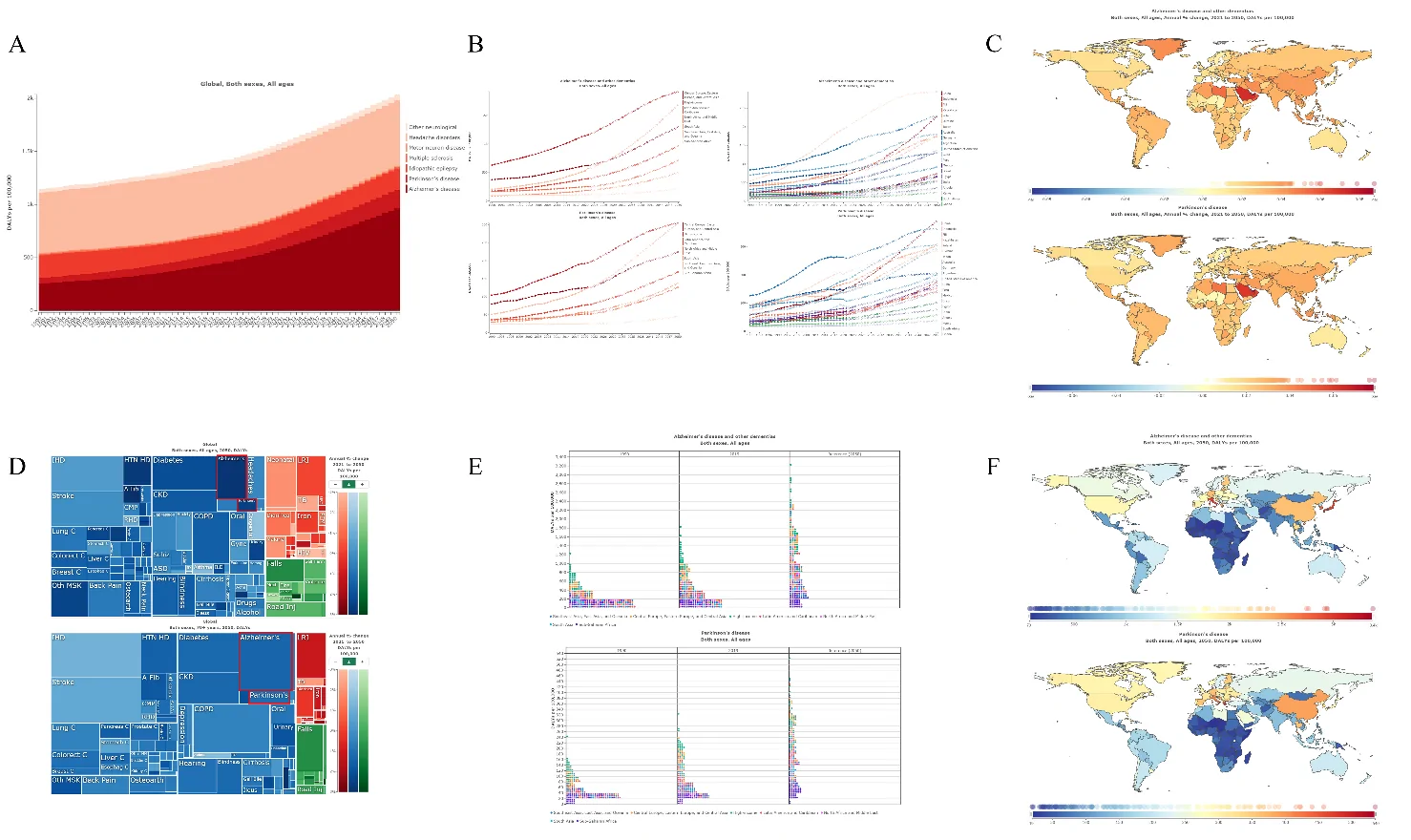
**Forecasts for global burden of neurodegenerative diseases, such as Alzheimer’s disease and other dementias, and Parkinson’s disease**. Foresights for trends of burdens to 2050 (A and B), annual changes from 2021 to 2050 (C), global burdens in 2050 (D and E), and geographical distributions in 2050 (F) on disability-adjusted life-years (DALYs) of neurodegenerative diseases such as Alzheimer’s disease and other dementias and Parkinson’s disease by Global Burden of Disease (GBD) studies 2021. Sub-Saharan Africa, Sub-Saharan Africa; S Asia, South Asia; MENA, North Africa, and the Middle East; SE & E Asia & Oceania, Southeast Asia, East Asia, and Oceania; Latin Am & Caribbean, Latin America and the Caribbean; C & E Europe & C Asia, Central Europe, Eastern Europe, and Central Asia; HI, high income. Regions were stratified based on the GBD super-regions, which group countries and territories that share similar geographic characteristics, epidemiological profiles, and causes of death. All panels were drawn via GBD Compare Data Visualization tools with data from GBD 2021 forecasts. *Source: Institute for Health Metrics and Evaluation, University of Washington. Used with permission. All rights reserved.*

Neurodegenerative diseases represent a growing public health concern, with their prevalence and impact on global health systems increasing significantly (Fig. 1) [11]. These conditions, which include AD and other dementias as well as PD, are characterized by the progressive degeneration of the nervous system, leading to a decline in cognitive and motor functions [24-28]. As the global population ages, the number of individuals affected by these diseases is expected to further increase in the future (Fig. 2), placing a substantial burden on healthcare resources, families, and societies [2, 29-33].

### 2.1 Alzheimer’s disease and other dementias

AD and other dementias represent a significant and growing proportion of the global burden of neurodegenerative diseases [31, 32]. According to the GBD 2021 study, the prevalence and impact of these conditions have continued to rise, driven by an aging population and improved life expectancy [4, 11]. The GBD 2021 data revealed that dementia, with AD being the most common form, is now one of the leading causes of disability and dependency among older adults [10, 11]. The GBD 2021 report indicates that the number of people living with AD and other dementia worldwide has increased substantially over the past few decades [11]. As of the latest estimates, approximately 21.8 million (19.1-24.8) people were living with dementia globally in 1990, and 56.9 million (49.4-65.0) million people were living with dementia in 2021. This stark increase underscores the urgent need for effective prevention, early diagnosis, and management strategies [34-36]. The global DALY count of dementia quickly increased from 13.6 million (6.4-29.6) in 1990 to 36.3 million (17.2-76.9) in 2021, increasing by approximately 168.7% (156.3-179.9) in the past three decades, with projections suggesting that this burden will continue to expand in the future [11, 37-39]. In terms of YLD, dementia accounts for a significant share, ranking as one of the top contributors to YLD in older age groups. The GBD 2021 study revealed that dementia was responsible for 4.4 million (3.0-5.8) YLDs in 1990 and 11.6 million (8.0-15.3) YLDs in 2021, reflecting not only the direct effects of cognitive decline but also the broader social and economic consequences of the disease [40-42]. These include the strain on healthcare systems, the need for long-term care, and the emotional and financial toll on families and caregivers [43, 44]. Moreover, the geographical distribution of dementia cases shows considerable variation, with higher rates observed in regions with more advanced demographic transitions, such as Europe and North America, which is related to their age structure being more skewed toward an aging population [11, 32, 45-47]. However, the fastest-growing populations of individuals with dementia are found in low- and middle-income countries (LMICs), where healthcare infrastructure may be less equipped to handle the increasing demand for services [17, 48, 49]. This disparity highlights the importance of tailored and culturally sensitive approaches to dementia care and support, as well as the need for increased investment in healthcare capacity and research in LMICs [50, 51].

Additionally, the GBD 2021 study revealed that while the majority of dementia cases are attributed to nonmodifiable risk factors such as age [52, 53] and genetics (such as the APOE, APP, PSEN1, and PSEN2 genes) [54-56], a significant portion can be linked to modifiable risk factors [20]. These include cardiovascular health, diabetes, smoking, and physical inactivity [20, 57-59]. Zhang *et al*. analyzed modifiable factors affecting dementia risk in the UK Biobank and revealed that lifestyle, medical history, and socioeconomic status contributed to dementia, and up to 47.0%-72.6% of dementia cases could be prevented [57]. Another two-sample Mendelian randomization study based on the UK Biobank by Sproviero *et al*. suggested that a causal effect exists between high blood pressure and a reduced late-life risk of AD [58]. A cross-sectional study of 46,011 Chinese adults aged 60 years or older suggested that old age, female sex, parental history of dementia, rural residence, fewer years of education, being widowed, being divorced, living alone, smoking, hypertension, diabetes, heart disease, and cerebrovascular disease were risk factors for dementia and mild cognitive impairment [59]. However, an individual-participant meta-analysis by Kivimäki *et al*. involving 404,840 people in 6.0 million person-years at risk suggested that increased dementia risk was observed in physically inactive individuals who developed cardiometabolic disease but was not significantly associated with all-cause dementia or AD, suggesting potential joint or mediation effects [60]. Public health interventions targeting these modifiable risk factors could reduce the incidence and delay the onset of dementia, thereby alleviating some of the burden on affected individuals and healthcare systems [61-63].

### 2.2 Parkinson’s disease

PD is another major neurodegenerative disorder that significantly contributes to the global burden of neurological diseases [2, 64, 65]. The GBD 2021 study provides critical insights into the current and projected impact of PD, highlighting its growing prevalence and associated health, social, and economic challenges [11, 64].

According to the GBD 2021, the number of people living with Parkinson’s disease has been steadily increasing, largely due to an aging population and increased life expectancy [11]. In 1990, approximately 3.1 million (2.7-3.6) people were considered to have PD, while it was estimated that approximately 11.8 million (10.4-13.4) individuals worldwide were affected by PD in 2021. The increasing incidence of PD underscores the need for more robust healthcare systems, improved diagnostic tools, and effective treatment options to manage the condition and support those affected [2, 11, 66]. The GBD 2021 data also revealed the substantial contribution of PD to the overall DALYs, which are a measure of the total years lost due to ill health, disability, or early death. PD was responsible for 1.9 (2.6-3.1) million DALYs and 0.5 million (0.3-0.6) YLDs in 1990 and increased to 7.5 (6.7-8.1) million DALYs and 1.7 million (1.2-2.2) YLDs in 2021 [11], indicating its significant impact on quality of life and the burden it places on both patients and their caregivers. This figure is expected to further increase as the world’s population continues to age [39, 67, 68]. The disease not only affects motor functions but also leads to nonmotor symptoms such as cognitive impairment, depression, and sleep disorders, further complicating patient care and management [3, 13, 26-28, 69-72]. Geographically, the burden of PD is distributed unevenly across the globe [11]. High-income countries, where the average age is relatively high, generally report a greater prevalence of PD [73-75]. However, similar to dementia cases, LMICs are experiencing a rapid increase in PD cases, partly due to demographic changes, and are more likely to be exposed to risk factors such as environmental toxins and diet [76-80]. These regions often face additional challenges, including limited access to specialized healthcare, medication, and rehabilitation services, which exacerbates the burden of the disease [77, 80]. Thus, the GBD 2021 findings emphasize the growing global burden of Parkinson’s disease, driven by an aging population and increasing prevalence in LMICs [11].

Moreover, the GBD 2021 highlights the importance of addressing modifiable risk factors for PD [20]. While the exact cause of PD remains unknown [26] and aging and genetic factors are inevitable [81-84], research suggests that certain environmental and lifestyle factors may play a role [85]. For example, exposure to pesticides, heavy metals, and other environmental toxins, as well as head trauma, has been linked to an increased risk of developing PD [85]. An umbrella review of meta-analyses by Bellou *et al*. indicated that physical activity and constipation presented Class I evidence for an association with PD and head injury, and anxiety or depression, beta-blockers, smoking, and serum uric acid presented Class II evidence for an association [85]. Another study by Knight *et al*. also suggested the role of diet and dietary patterns in the risk of PD or alleviating PD severity [86]. Modifiable lifestyle factors such as physical activity and tea and coffee consumption may reduce the risk of PD [87-89]. Public health strategies aimed at reducing these exposures, along with promoting healthy lifestyles, could lower the incidence of PD and mitigate its impact [90]. To address this challenge effectively, there is a need for enhanced public health initiatives, improved access to care, and continued research into the underlying causes and potential treatments for PD [91].

## 3. Unmet medical and social needs

The increasing prevalence of neurodegenerative diseases has exposed significant gaps in the current medical and social support systems. With global aging trends, the urgent crisis and challenges in nervous system health loss caused by neurodegenerative diseases should be highlighted, which is a public health priority [6, 92, 93]. However, owing to the increasing global burden, access to essential medications is not available for a large proportion of patients with neurodegenerative diseases, especially those living in LMICs [6, 16, 17]. Although promising novel therapeutic options exist [94-96], many neurodegenerative diseases have not been verified with widely available clinically proven curative treatments [91, 96]. These unmet needs are multifaceted and encompass a range of issues that affect both patients and their caregivers [13, 14]. Addressing these gaps is crucial for improving the quality of life, care, and equity for those affected by these conditions [5, 6, 97, 98].

### 3.1. Access to Specialized Care and Services

One of the most pressing unmet needs is the lack of access to specialized healthcare providers and services [99-101]. Many regions, particularly LMICs, have a shortage of neurologists, geriatricians, and other specialists trained in the diagnosis and management of neurodegenerative diseases [17, 48, 49, 77, 80]. This scarcity often results in delayed or inaccurate diagnoses, which can lead to suboptimal treatment and management [102-104]. Additionally, there is a need for more comprehensive and integrated care models that include multidisciplinary teams, such as neurologists, neurosurgeons (for emerging neuromodulation, especially deep brain stimulation therapy [94, 105-107]), psychiatrists, physical therapists, occupational therapists, and social workers, to provide holistic and coordinated care [108-110].

### 3.2. Early Diagnosis and Intervention

Early diagnosis and intervention are critical for managing neurodegenerative diseases effectively [111-113]. However, many individuals do not receive a timely diagnosis because of a lack of awareness, stigma, and limited availability of diagnostic tools and services [103, 114]. Early detection allows for earlier initiation of treatments, which can help manage symptoms and potentially slow the progression of the disease [115]. There is a need for more widespread screening programs, public education campaigns, and improved access to diagnostic technologies, such as advanced imaging and biomarker testing, to facilitate early identification and intervention [116-119].

### 3.3. Support for Caregivers

Caregivers play a vital role in the lives of individuals with neurodegenerative diseases, but they often face significant emotional, physical, and financial burdens [13, 14, 120]. Many caregivers lack the necessary training, resources, and support to manage the complex needs of their loved ones [121, 122]. There is a need for increased support services, including respite care, counseling, and caregiver training programs, to help alleviate the strain on caregivers and improve their well-being [122, 123]. Additionally, policies that recognize and support the role of informal caregivers, such as flexible work arrangements and financial assistance, are essential [124, 125].

### 3.4. Long-Term Care and Community Integration

As neurodegenerative diseases progress, many individuals require long-term care, which can be challenging to access and afford [126, 127]. There is a need for more affordable and accessible long-term care options, including in-home care, assisted living facilities, and nursing homes [122, 128]. Furthermore, there is a growing recognition of the importance of community integration and the need for age-friendly and dementia-friendly communities [15, 93, 126]. These environments should provide safe, supportive, and inclusive spaces that allow individuals with neurodegenerative diseases to remain engaged and active members of their communities [5].

### 3.5. Research and Innovation

Despite significant advances in research, there is a lack of effective treatments and cures for many neurodegenerative diseases [1, 47, 91]. Increased investment in research is needed to better understand the underlying mechanisms of these diseases, develop new therapeutic approaches, and improve existing treatments [129-131]. Additionally, there is a need for more translational research to bridge the gap between scientific discoveries and clinical applications [132]. Public-private partnerships, international collaborations, and patient-centered research initiatives can help accelerate progress in this area [5].

### 3.6. Policy and Advocacy

Effective policy and advocacy are essential for addressing the unmet needs of individuals with neurodegenerative diseases [6, 126]. Policies should aim to improve access to care, reduce healthcare disparities, and ensure that the needs of this population are considered in broader health and social policies [99, 133]. Advocacy efforts should focus on raising awareness, reducing stigma, and promoting the rights and dignity of individuals with neurodegenerative diseases and their caregivers [134].

## 4. Challenges during and after the COVID-19 pandemic

The COVID-19 pandemic has presented unprecedented challenges for individuals with neurodegenerative diseases, their caregivers, and the healthcare systems that support them [8, 9, 135]. The global health crisis has not only exacerbated existing issues but also introduced new and complex obstacles that have significantly impacted the care and well-being of this vulnerable population [21, 136, 137]. The COVID-19 pandemic could worsen or even trigger neurological symptoms, and lockdown policies in different countries/regions might increase the difficulty of accessing medications, resulting in increased burdens [10, 138, 139]. Even after the pandemic, the long-term effects and recurrent infection of COVID-19 could also represent a potential crisis, especially for the aging population with neurodegenerative diseases [140].

### 4.1. Increased Health Risks and Mortality

With the widespread spread of the COVID-19 pandemic, emerging evidence has suggested links between COVID-19 and neurodegenerative diseases [21, 136, 141, 142]. More seriously, COVID-19 infection might be considered responsible for the potential causes of symptom worsening, disease progression, and management challenges associated with PD and dementia [21, 136, 141, 142]. The potential links between COVID-19 and neurodegenerative diseases may be complicated [21, 142], but there are clear clues that patients infected with COVID-19 have worsened or even triggered motor and nonmotor symptoms and experienced increased mortality rates [143, 144]. Although the WHO announced the end of the global emergence of COVID-19, its long-term effects, especially neurological symptoms, and reinfections, could still represent a global health crisis for patients [145]. In addition, individuals with neurodegenerative diseases, such as AD and other dementias, as well as PD, are at increased risk of severe complications from COVID-19 [21, 137]. The GBD 2021 and other studies have shown that these patients, particularly those in long-term care facilities, are more susceptible to severe illness, hospitalization, and death [8, 146, 147]. This increased vulnerability is due to several factors, including advanced age, comorbidities, and the physical and cognitive impairments associated with neurodegenerative conditions, which can make it difficult to follow public health guidelines and self-protect against the virus [9, 148, 149].

### 4.2. Disruption of Healthcare Services

The pandemic has led to significant disruptions in healthcare services, affecting the diagnosis, treatment, and management of neurodegenerative diseases [135]. Many non-urgent medical appointments, diagnostic procedures, and elective surgeries (such as neuromodulation surgeries) have been postponed or canceled to redirect resources to the pandemic response [94, 150, 151]. This delay in accessing timely medical care has several consequences, including the progression of symptoms, delayed diagnoses, and missed opportunities for early intervention [135]. During the COVID-19 pandemic, the medical, surgical, and physical management of neurodegenerative diseases could also be challenging because of the lockdown policies of different countries/regions, which prevent some patients from receiving convenient and timely treatments [94, 151]. Remote monitoring and management are accessible and efficient for patients, providing lessons and experience for interpersonal isolation during pandemics and alleviating crises to a certain degree [152-155]. However, the shift to remote and telemedicine, while beneficial in some cases, has posed challenges for patients with cognitive (especially dementia) and technological limitations [155].

### 4.3. Isolation and Mental Health Impact

Social distancing measures and lockdowns have led to increased isolation and reduced social interaction, which have had a profound impact on the mental health of individuals with neurodegenerative diseases and their caregivers [156-158]. For people with dementia, the lack of routines and structure, which are essential for their well-being, has increased behavioral and psychological symptoms, such as agitation, anxiety, and depression [159, 160]. Caregivers have also experienced heightened stress, fear, and uncertainty, leading to increased rates of caregiver burnout and mental health issues [161, 162]. The need for social and emotional support has become even more critical during these times and has shown potential as a long-term effect of consecutive COVID-19 waves [163].

### 4.4. Economic and Financial Strain

The economic downturn and job losses resulting from the pandemic have placed additional financial strain on families caring for individuals with neurodegenerative diseases. Many caregivers, especially women, have had to leave the workforce to provide care at home, leading to long-term economic consequences and potentially widening gender disparities. The increased cost of providing care, coupled with the financial impact of the pandemic, has created significant economic challenges for many families.

### 4.5. Long-term Care Facilities and Infection Control

Long-term care facilities, which house a large proportion of individuals with neurodegenerative diseases, have been epicenters of COVID-19 outbreaks [164, 165]. The close living quarters, shared spaces, and presence of vulnerable residents have made these facilities particularly susceptible to the rapid spread of the virus [165]. Infection control measures, such as visitor restrictions and quarantine protocols, have been necessary but have also contributed to the isolation and distress of patients and their families [166, 167]. Ensuring the safety and well-being of residents in these settings has required significant efforts and resources.

### 4.6. Adaptation and Resilience

Despite these challenges, the pandemic has also spurred innovation and adaptation in the care of individuals with neurodegenerative diseases. Telemedicine and virtual support services have become more widely accepted and utilized, providing a means to deliver care and support while adhering to social distancing guidelines [152-155]. Community organizations and healthcare providers have developed new programs and resources to address the unique needs of this population during the pandemic, demonstrating resilience and a commitment to ongoing support [168]. These adaptations have the potential to improve care delivery and support structures in the long term.

In summary, the COVID-19 pandemic has highlighted and exacerbated the vulnerabilities of individuals with neurodegenerative diseases and their caregivers. Addressing these challenges requires a multifaceted approach, including the restoration and enhancement of healthcare services, the provision of mental health support, the facilitation of timely medical care, and the development of resilient and adaptable healthcare systems capable of responding to future crises. By doing so, we can better support the well-being of those affected by neurodegenerative diseases and their caregivers, both during and after the pandemic.

## 5. Future improvements in healthcare policy and practice

Concerning healthy aging, many more timely health services and policy implications are still warranted for the unmet medical and social needs of individuals with neurodegenerative diseases in preparation for an aging world [5, 7, 97, 169]. To address the growing burden of neurodegenerative diseases effectively and ensure that patients receive the necessary care and support, several improvements can be made in both healthcare policy and practice [5, 17, 99]. These improvements aim to better equip the healthcare system to manage the increasing prevalence of neurodegenerative diseases while also preparing for future global health challenges.

### 5.1. Develop International Partnerships for Medication Distribution

International partnerships should be established to improve the distribution of essential medications to low-income countries [170]. This includes ensuring that life-saving and symptom-managing drugs are accessible and affordable, even in regions with limited resources [171]. Collaborations between governments, nongovernmental organizations (NGOs), and pharmaceutical companies can facilitate the equitable distribution of these critical medications.

### 5.2. Implement Subsidy Programs

Subsidy programs should be introduced to make treatments more affordable for underprivileged populations. Financial barriers often prevent individuals from accessing the medications and therapies they need [172]. By providing subsidies or implementing tiered pricing systems, healthcare systems can ensure that all individuals, regardless of their economic status, have access to the necessary treatments [173, 174].

### 5.3. Invest in Healthcare Infrastructure

Significant investment is needed to strengthen healthcare infrastructure, particularly in regions with inadequate medical facilities. This includes building and upgrading hospitals, clinics, and long-term care facilities, as well as equipping them with the necessary diagnostic and treatment tools [19]. An improved infrastructure will increase the capacity of healthcare systems to provide high-quality care to a growing number of patients with neurodegenerative diseases [99].

### 5.4. Train Healthcare Professionals

There is a need to train more healthcare professionals in the management of neurodegenerative diseases. Specialized training programs for doctors, nurses, and allied health professionals can ensure that expert care is widely available [16, 19]. This includes education on the latest diagnostic techniques, treatment protocols, and best practices in patient-centered care [122].

### 5.5. Increase Research Funding

Increased funding for research into neurodegenerative diseases is crucial for discovering new treatments and potentially curative therapies. Investment in basic, translational, and clinical research can lead to breakthroughs in understanding the underlying mechanisms of these diseases and developing more effective interventions [5, 16, 126]. Public and private funding sources should be leveraged to support this vital research.

### 5.6. Encourage Public-Private Partnerships

Public-private partnerships can accelerate the development of innovative therapies. Collaboration among government agencies, academic institutions, and private sector entities can drive innovation and bring new treatments to the market more quickly [5, 126]. Such partnerships can also help in scaling up successful interventions and making them more widely available.

### 5.7. Create Awareness Campaigns

Awareness campaigns should be launched to educate the public and policymakers about the impact of neurodegenerative diseases and the importance of timely intervention [175]. Advocacy efforts should focus on raising awareness, reducing stigma, and promoting the rights and dignity of individuals with these conditions [98, 175]. Policymakers should be encouraged to prioritize the health needs of the aging population through supportive policies and legislation.

### 5.8. Develop Comprehensive Care Plans

Comprehensive care plans that include physical, emotional, and social support should be developed for patients with neurodegenerative diseases [122]. These plans should be tailored to the individual needs of each patient and should involve a team of healthcare providers [108, 176]. Community support programs should also be established to assist families and caregivers, providing them with the resources and support they need to manage the day-to-day challenges of caring for a loved one with a neurodegenerative disease.

### 5.9. Formulate Contingency Plans

Healthcare systems should formulate contingency plans to ensure continuous access to medications and care during lockdowns or similar disruptions. The lessons learned from the COVID-19 pandemic should inform these plans, with a focus on maintaining essential services and minimizing the impact on vulnerable populations [135, 154]. Additionally, there should be a concerted effort to study the long-term effects of COVID-19 on neurological health to better prepare for future healthcare needs.

### 5.10. Foster International Collaborations

International collaborations should be fostered to share data, resources, and best practices in managing neurodegenerative diseases. Participation in global health initiatives that aim to address the challenges posed by an aging population is essential. By working together, countries can learn from each other’s experiences, pool resources, and develop more effective strategies to combat these debilitating conditions [177-179]. To address the growing burden of neurodegenerative diseases, a coordinated and multifaceted approach is needed. By implementing these suggested improvements in healthcare policy and practice, we can create a more resilient and responsive healthcare system that provides the necessary care and support for individuals with neurodegenerative diseases, their families, and caregivers.

## 6. Conclusion

The increasing global burden of neurodegenerative diseases, such as Alzheimer’s disease, other dementias, and Parkinson’s disease, presents a significant challenge to healthcare systems, societies, and individuals worldwide. The demographic shift toward an aging population, coupled with the ongoing impacts of the COVID-19 pandemic, has highlighted the urgent need for comprehensive and coordinated efforts to address the unmet medical and social needs of those affected by these conditions.

To effectively manage the increasing burden, several key improvements in healthcare policy and practice are essential. These include developing international partnerships for medication distribution, implementing subsidy programs to make treatments more affordable, investing in healthcare infrastructure, training more healthcare professionals, and increasing funding for research. Additionally, fostering public-private partnerships, creating awareness campaigns, and formulating comprehensive care plans that integrate physical, emotional, and social support are crucial steps. Contingency planning for future disruptions and fostering international collaborations to share best practices are also vital.

By addressing these unmet needs and implementing the suggested improvements, we can better equip healthcare systems to provide high-quality, accessible, and supportive care. This not only enhances the quality of life for individuals with neurodegenerative diseases and their caregivers but also contributes to the overall resilience and sustainability of healthcare systems. Policymakers, healthcare providers, researchers, and communities must work together to create a more inclusive and supportive environment for those affected by neurodegenerative diseases, ensuring that they receive the care and support they need now and in the future.

## Data Availability

All panels were drawn via GBD Compare Data Visualization tools from data from GBD 2021 estimates. More information is available at the Institute for Health Metrics and Evaluation (IHME), University of Washington: http://www.healthdata.org.
